# Gemcitabine and docetaxel in relapsed and unresectable high-grade osteosarcoma and spindle cell sarcoma of bone

**DOI:** 10.1186/s12885-016-2312-3

**Published:** 2016-04-20

**Authors:** E. Palmerini, R. L. Jones, E. Marchesi, A. Paioli, M. Cesari, A. Longhi, C. Meazza, L. Coccoli, F. Fagioli, S. Asaftei, G. Grignani, A. Tamburini, S. M. Pollack, P. Picci, S. Ferrari

**Affiliations:** PROMETEO Laboratory/Chemotherapy Unit, Istituto Ortopedico Rizzoli, Bologna, Italy; University of Washington/ Fred Hutchinson Cancer Research Center, Seattle, USA; Oncoematologia Pediatrica INT, Milan, Italy; UO Oncoematologia Pediatrica Pisa, Pisa, Italy; Oncoematologia Pediatrica Torino, Torino, Italy; IRCCS Candiolo, Torino, Italy; Oncoematologia Azienda Ospedaliera Universitaria Meyer Pediatrica, Firenze, Italy; Laboratory Research, Istituto Ortopedico Rizzoli, Bologna, Italy; PROMETEO Laboratory/Section of Chemotherapy, Research, Innovation & Technology (RIT) Department, Istituto Ortopedico Rizzoli, Via Pupilli, 1, 40136 Bologna, Italy

**Keywords:** Osteosarcoma, High-grade bone sarcoma, Gemcitabine, Docetaxel, Chemotherapy

## Abstract

**Background:**

Few new compounds are available for relapsed osteosarcoma. We retrospectively evaluated the activity of gemcitabine (G) plus docetaxel (D) in patients with relapsed high-grade osteosarcoma and high-grade spindle cell sarcoma of bone (HGS).

**Methods:**

Patients receiving G 900 mg/m^2^ d 1, 8; D 75 mg/m^2^ d 8, every 21 days were eligible. Primary end-point: progression-free survival (PFS) at 4 months; secondary end-point: overall survival (OS) and response rate.

**Results:**

Fifty-one patients were included, with a median age of 17 years (8–71), 26 (51 %) were pediatric patients. GD line of treatment: 2nd in 14 patients, ≥3rd in 37. 25 (49 %) patients had metastases limited to lungs, 26 (51 %) multiple sites. Histology: 40 (78 %) osteosarcoma, 11 (22 %) HGS. Eight (16 %) patients achieved surgical complete response (sCR2) after GD.

Four-month PFS rate was 46 %, and significantly better for patients with ECOG 0 (ECOG 0: 54 % vs ECOG 1: 43 % vs ECOG 2: 0 %; *p* = 0.003), for patients undergoing metastasectomy after GD (sCR2 75 % vs no-sCR2 40 %, *p* = 0.02) and for osteosarcoma (osteosarcoma 56 % vs HGS 18 %; *p* = 0.05), with no differences according to age, line of treatment, and pattern of metastases.

Forty-six cases had RECIST measurable disease: 6 (13 %) patients had a partial response (PR), 20 (43 %) had stable disease (SD) and 20 (43 %) had progressive disease (PD).

The 1-year OS was 30 %: 67 % for PR, 54 % for SD and 20 % for PD (*p* = 0.005).

**Conclusions:**

GD is an active treatment for relapsed high-grade osteosarcoma, especially for ECOG 0 patients, and should be included in the therapeutic armamentarium of metastatic osteosarcoma.

## Background

At present, patients with nonmetastatic osteosarcoma of the extremity under the age of 40 years, have an expected 5-year survival rate of 70 % with multi-modality management consisting of chemotherapy (based on methotrexate, cisplatin, doxorubicin and ifosfamide) and surgery [1, 2].

While the outcome of patients with localized osteosarcoma of bone has improved with the introduction of multi-agent chemotherapy in combination with surgery [1, 2], treatment options for patients with relapsed disease are limited and post-relapse survival is poor, with a 5-year post relapse survival (PR) rate below 30 % [3].

The role of second-line chemotherapy for recurrent osteosarcoma is much less well defined, and there is no accepted standard regimen. Treatment choice may take into account the prior disease-free interval, and often includes ifosfamide ± etoposide ± carboplatin, and other active drugs [4].

High-dose ifosfamide (HDIFO) has been widely used for patients with metastatic osteosarcoma [5, 6], but, no new drugs were FDA or EMA approved over the last 25 years.

Prospective trials with agents such as pemetrexed or sorafenib and sorafenib/everolimus were performed [7–9]. Some of these agents have shown modest activity in osteosarcoma, but none were deemed worthy of further development.

In general, there are few indications for radiation therapy, but there are anatomical locations in which the possibility of complete surgical resection is limited. In these cases, radiation may be an option to try to extend the progression-free interval. Novel local treatment techniques (e.g. proton beam therapy, radiofrequency ablation and isolated limb perfusion) may have a role in specific patients, under the management of a multi disciplinary team [4].

The combination of gemcitabine (G) plus docetaxel (D) is active in soft tissue sarcomas, with published data indicating higher activity than gemcitabine alone [10–12]. Although the biology of soft tissue sarcomas is fundamentally different from that of bone sarcoma, the efficacy of these two drugs has also been investigated in patients with recurrent osteosarcoma with conflicting results (Table 1; [13–18]).

**Table 1:** Gemcitabine and docetaxel in advanced osteosarcoma

Drugs	Pts n.	RR	CR/PR	Authors
G 1,000 mg/m2	7	0 %	0/0	Merimsky O, Sarcoma 2000 [13]
G 675 mg/m2 D 75–100 mg/m2	10	30 %	0/3	Navid F, Cancer 2008 [14]
G 900 mg/m2 D 80–100 mg/m2	14	7 %	0/1	Fox E. SARC 003, Oncologist 2010 [15]
G 675 mg/m2 ± D 75–100 mg/m2	4	25 %	0/1	Gosiengfiao Y, J Pediatr Hematol Oncol 2012 [16]
G 675 mg/m2 + D 75–100 mg/m2	18	5 %	0/1	Qi WX, Jpn J Clin Oncol 2012 [17]
G 675–900 mg/m2 D 100 mg/m2	17	24 %	3/1	Song BS, Pediatr Blood Cancer 2014 [18]

*GD* gemcitabine and docetaxel, *RR* response rate, *CR* complete response, *PR* partial response

Here we report the results of a retrospective multicenter analysis of the activity of this combination in patients with recurrent high-grade osteosarcoma and high-grade spindle cell sarcoma of bone (HGS).

This analysis involves both pediatric and adult patients, primarily as management for patients with localized disease is the same, regardless of age.

## Methods

A joint analysis between the Italian Sarcoma Group and the Sarcoma Center of the University of Washington was planned, in order to collect data on patients with metastatic high grade bone sarcomas treated with GD.

This study was approved by the ethics committees of all 6 centers participating to the study: 5 Italian referral centers (Rizzoli Institute, Bologna; Tumor National Institute, Milan; Pediatric Oncology Departments of Turin; Meyer Children’s Hospital, Florence; Pediatric Onco-Hematology, Pisa) and the Sarcoma Center of the University of Washington.

All patients included in the study signed informed consent for treatment and privacy according to each individual institution’s requirements.

The analysis period was set from January 2012 and August 2014.

Patients with the following characteristics were included: 1) diagnosis of high-grade osteosarcoma and spindle cell sarcoma of bone, 2) recurrent or advanced disease not amenable of surgical treatment, 3) disease progression after at least one line of chemotherapy, 4) treatment with GD, 5) availability of demographic, clinical and follow-up data. The following were required for cases evaluable for response 1) treated with at least 2 cycles of GD, 2) having measurable disease as per RECIST 1.1 and 3) with radiological images for review.

The diagnosis was confirmed in all cases by an experienced bone sarcoma pathologist.

Drugs were administered as follows: G 675–900 mg/m^2^ over 90 min on Day 1 and G 675–900 mg/m^2^ and D 75 mg/m^2^ on Day 8. All patients received pre-medication with steroids prior to docetaxel.

Patient characteristics including age, gender, ECOG performance status, primary tumor site, site and number of metastatic lesions, type and number of prior treatments, response to therapy, toxicity, date of progression, date of last follow-up or death were obtained from the databases or the patient clinical chart and collected in a study-specific case report form.

All relevant radiological images we re-reviewed for the purpose of this study (EP, RLJ). Response was assessed using the Response Evaluation Criteria In Solid Tumors (RECIST) version 1.1. Patients were assessed for response after the first 2 cycles and, in case of response or stable disease, every 2 or 3 cycles of therapy. Objective response was expressed as response rate (complete response [CR] + partial response [PR]), stable disease (SD) or progressive disease (PD).

Toxicity data were collected from clinical chart and from “patient-toxicity” questionnaires, in some of the centers. Toxicity was graded according to the Common Toxicity Criteria for Adverse Events (CTCAE) version 4. In case of grade 4 neutropenia prophylactic use of G-CSF was allowed; therapeutic use of G-CSF was mandatory in case of febrile neutropenia.

Treatment was discontinued at progression or unacceptable toxicity. All patients who received at least one cycle (one cycle was defined as G on Day 1 and GD on Day 8, every 21 days) were included in an intention-to-treat analysis.

Progression-Free Survival (PFS) and Overall Survival (OS), were estimated according to the Kaplan and Meier method with their respective 95 % confidence intervals (CI) and calculated from the first day of chemotherapy administration to tumor progression (PFS) or death or last follow-up visit (OS).

Metastasectomy was performed on a “case-by-case” basis, following multidisciplinary discussion. Only patients with confirmed response (partial response or stable disease after 2 consecutive assessments) were considered for surgical removal of metastases. If excision of all secondary lesions became possible, patients were classified as achieving a second surgical complete remission (sCR2).

## Results

Fifty-one patients were included in the study. The clinical characteristics are shown in Table 2. Twenty-six patients (51 %) were aged less than 18 years. Most of the patients had an ECOG performance status of 0 and 73 % had received 1 or more chemotherapy lines (with a maximum of 5 lines). 40 (78 %) patients had high-grade osteosarcoma and 11 (22 %) had HGS. The median age was 17 years (range 8 to 71 years): 14.5 years (range 8 to 59) for osteosarcoma patients and 36 years (range 18 to 71) for the 11 patients with HGS.

**Table 2:** Clinical, Pathologic and Treatment Variable patients with osteosarcoma and high grade spindle cell sarcoma of bone

Characteristics	Pts n.	%
Histology		
Osteosarcoma	40	78 %
HGS	11	22 %
Gender		
Male	36	70 %
Female	15	30 %
Age		
≥18 years	25	49 %
<18 years	26	51 %
ECOG		
0	33	64 %
1	14	28 %
2	4	8 %
Line of CT for metastases		
1	14	27 %
2	28	55 %
≥3	9	18 %
Pattern of metastases		
Lung Only	25	49 %
Multiple	26	51 %
Bone metastases		
Yes	13	25 %
No	38	75 %

*HGS* High grade spindle cell sarcoma of bone, *GD* gemcitabine and docetaxel, *CT* chemotherapy

All patients had received neoadjuvant/adjuvant chemotherapy with doxorubicin (cumulative dose 360–420 mg/m^2^), cisplatin (600 mg/m^2^), ifosfamide (30–60 gr/m^2^), while methotrexate (36–60 gr/m^2^), was administered to all patients younger than 40 years.

Fourteen (27 %) patients received GD at their first recurrence. Thirty-seven patients received GD combination after failure of prior chemotherapy lines (with a maximum of 5 lines) (Table 2). HDIFO (ifosfamide 15 g/m^2^ plus mesna as a 5 day-continuous infusion or 14 gr/m^2^ in 14 day-continuous infusion) was offered to all cases prior to GD: in 1st line, or in the adjuvant setting in case of poor response to neoadjuvant chemotherapy. Other drugs employed in the metastatic setting were: cyclophosphamide and etoposide, ifosfamide and etoposide, sorafenib, sorafenib and everolimus, pemetrexed, vinorelbine and anti IGF-1R based therapies.

The schedule of gemcitabine at a lower dose (675 mg/m^2^) was employed in 8 cases, mainly in pediatric patients.

### Response assessment

Five patients were excluded from the response evaluation: two patients did not receive docetaxel due to an allergic reaction, one for grade 4 skin toxicity after the first cycle and 2 patients with metastatic lesions that were non measurable by RECIST.

**Fig. 1:**
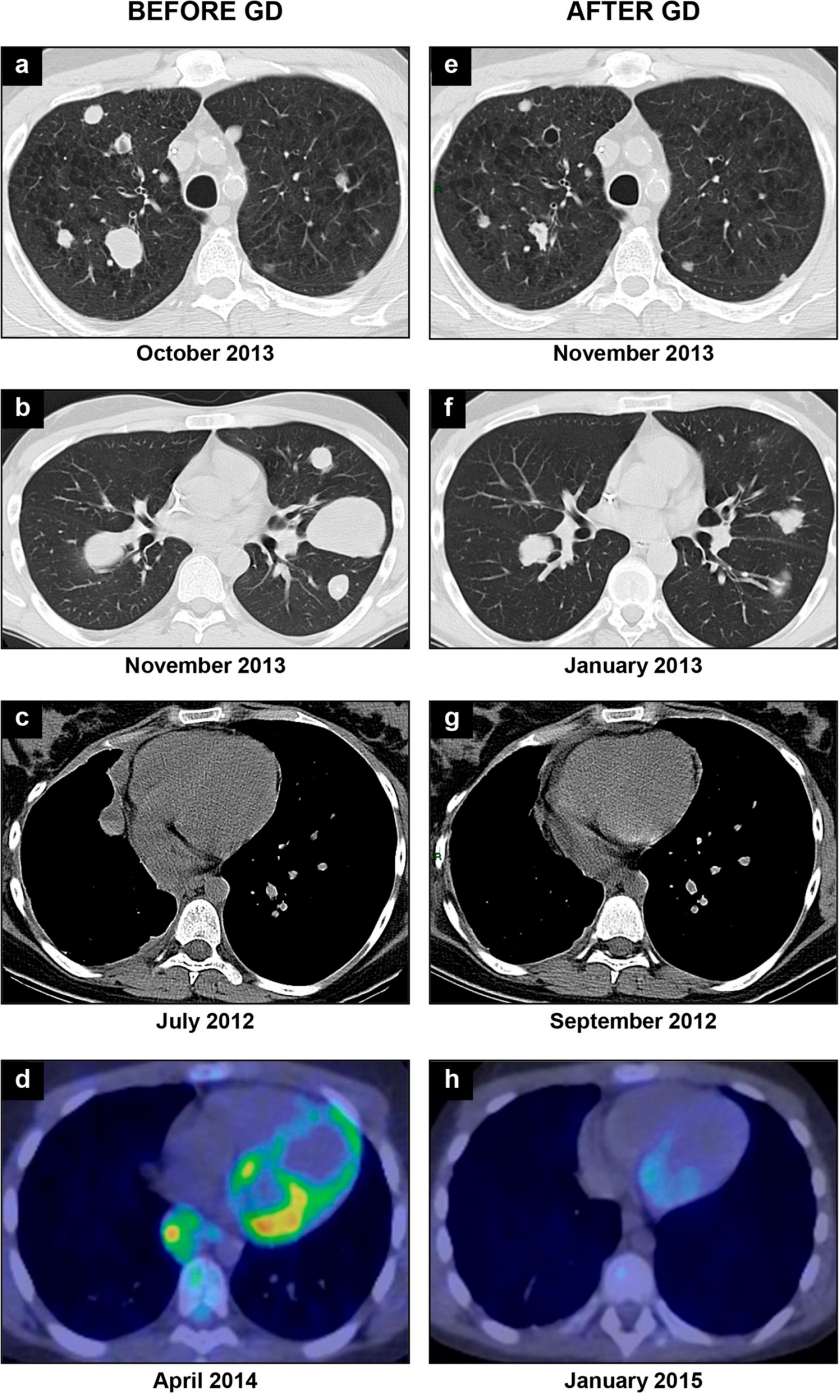
RECIST responses by chest CT scan in 3 patients with bilateral lung metastases, before gemcitabine and docetaxel (GD) treatment (**a**, **b**, **c**) and after 2 cycles of therapy (**e**, **f**, **g**); PET/CT imaging showing 18 F-FDG uptake in a patient with osteosarcoma lung metastastases, before GD treatment (**d**) and after 7 cycles of therapy (**h**)

In 46 patients evaluable for response: 6 patients achieved a PR (6/46 [13 %]) and 20 (43 %) SD, with a median duration of response lasting 6.5 months (range 2–11) for PRs and 4 months (range 2 to 16 months), for patients with SD. All patients achieving a partial response had classic osteosarcoma histology, and 5 of them were pediatric. Twenty out of 46 patients experienced progressive disease (PD) (20/46, 43 %) (Fig. 1, Table 3).

**Table 3:** Grade 3-4 non-hemathologic toxicity in 51 patient receiving gemcitabine and docetaxel (GD)

G3-4 non-hemathologic toxicity	Compliance	Outcome	GD cys
Allergic reaction (1st cycle)	Stop docetaxel	Resolved	2
Allergic reaction (1st cycle)	Stop docetaxel	Resolved	4
Allergic reaction (4th cycle)	Stop docetaxel	Resolved	5
Capillary leak syndrome	Stop docetaxel	Resolved	9
Diarrhea	Delay/dose reduction	Resolved	6
Febrile neutropenia	Delay	Resolved	7
Lung fibrosis	None	Resolved	5
Foot syndrome	None	Resolved	2
Stephen Johnson syndrome	Stop docetaxel	Off -treatment	1

*GD cys* gemcitabine and docetaxel number of chemoteherapy cycles

### Toxicity

The safety analysis is based on the 51 patients who received at least one dose of chemotherapy. The median number of cycles administered was 4 (range 1–20 cycles). Grade 4 hematological toxicity was observed in 13 (25 %) patients, with 11 (21 %) experiencing grade 4 neutropenia and two grade 4 thrombocytopenia. Non-hematological toxicity was reported in 8 (16 %) patients, with 3 (6 %) patients experiencing allergic reactions (two following the first cycle and one after 4 cycles). Diarrhea (grade 1) was reported in 2 cases, lung fibrosis, Steven Johnson syndrome and capillary leak syndrome in one patient each (Table 4).

**Table 4:** Responses according to histology in 46 patients with measurable disease by RECIST

	Osteosarcoma (*n* = 35) *n* (%)	HGS (*n* = 11) *n* (%)	All (46) *n* (%)
PR	6 (17)	0	6 (13)
SD	14 (40)	6 (55)	20 (43)
PD	15 (43)	5 (45)	20 (43)

*HGS* High grade spindle cell sarcoma of bone

No differences of toxicity were observed according to G dose.

### Outcome

#### Progression free survival

**Fig. 2:**
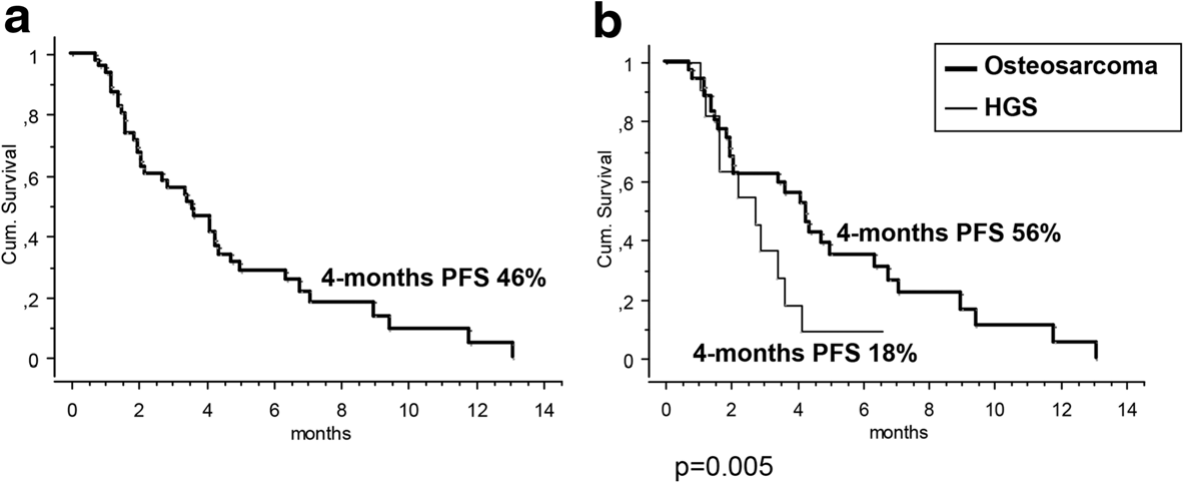
Progression-free survival curves at 4 months (4-month PFS) in all 51 patients (**a**) and by histology (**b**): high-grade osteosarcoma (40 pts) and high-grade spindle cell sarcoma of bone (HGS; 11 pts)

The median PFS was 3.5 months (range 1–13.5 months). The 4- and 6-month PFS were 46 % (95 % CI; 31–61 %) and 28 % (95 % CI, 15–42), respectively (Fig. 2a, Table 5). The 4-month PFS was significantly better for patients with osteosarcoma compared to HGS (Fig. 2b). No significant difference was observed according to age, gender, number of lines of systemic therapy and pattern of metastatic spread. Patients with a good performance status (ECOG 0) had significantly better PFS (Table 5).

**Table 5:** Univariate Analysis of Clinical, Pathologic and Treatment Variable for PFS in patients with osteosarcoma and high grade spindle cell sarcoma of bone

Characteristics	Pts n.	% 4-months PFS	95 % CI	*P*-value
Overall				
	46	46	31–61	
Age				
≥18 years	23	65	43–88	0.4
<18 years	23	35	17–53	
Sex				
Female	12	50	22–78	0.7
Male	34	44	27–62	
Mets Site				
Lung only	24	61	41–81	0.1
Multiple	22	30	11–50	
Line GD				
2	13	60	32–87	0.4
≥3	33	41	24.58	
ECOG				
0	29	54	35–72	0.003
1	13	43	15–71	
2	4	0	0	
Histology				
Osteosarcoma	35	56	39–73	0.005
HGS	11	18	0–41	

*PFS* Progression Free Survival, *HGS* high grade spindle cell sarcoma of bone, *GD* gemcitabine and docetaxel

### Overall survival post GD

The median survival time post gemcitabine plus docetaxel was 7.5 months (range 2–45 months).

The 1-year overall survival was 30 % (95 % CI; 22–57). For patients achieving a PR the 1-year OS was 67 % (95 % CI; 13–100), for those with SD it was 54 % (95 % CI; 25–85), and for those with PD 20 % (95 % CI; 10–38) (*p* = 0.005).

**Fig. 3:**
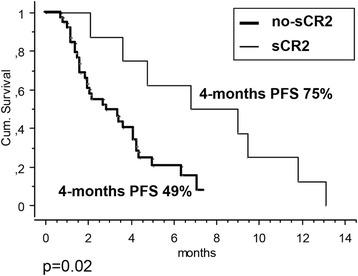
Progression-free survival curves at 4 months (4-month PFS) in eight patients rendered macroscopically free of disease following chemotherapy and metastasectomy (2sCR) versus 4-month PFS in the remaining 43 patients (no-2CR2); 2sCR: surgical complete remission

Following GD, 8 patients underwent surgery and achieved a surgical complete response (sCR2). Six of these patients had lung metastases, 1 had a gastric lesion and another patient had a local recurrence with a synchronous subcutaneous metastasis. All these patients had osteosarcoma and the median age was 14.5 years (range 11–23 years). The 4-month PFS for patients achieving sCR2 was 75 % (95 % CI; 45–100), and 49 % (95 % CI; 24–56) for the others, *p* = 0.02 (Fig. 3).

## Discussion

Our multi-institutional retrospective study evaluated the activity of GD in a group of heavily pretreated patients with high grade osteosarcomas and HGS.

Our study demonstrated a 4-month PFS of 46 %, with better PFS in patients with a good performance status. Also, histology seems an important predictor of response, with a 4-month PFS of 56 % in patients with classic high-grade osteosarcoma, as compared to a 4-month PFS of only 18 % for HGS. Of note, all pediatric patients had a diagnosis of classic osteosarcoma. These findings were confirmed in terms of response rate, with partial responses observed only in patients with high-grade osteosarcoma, whereas no objective response was seen in the HGS cohort, suggesting that patients with recurrent HGS should be offered participation in clinical trials of novel agents.

The PFS observed in the present series (46 % at 4 months) is superior to the 4-month PFS value of 40 %, the threshold to identify active agents in advanced soft tissue sarcomas set by Van Glabbeke and colleagues [19]. It is also superior to the PFS of a similar group of patients treated in a prospective Phase II study with pemetrexed (3-month PFS 17.2 % (95 % CI: 3.5–31) [7], and equivalent to that of sorafenib (4-month PFS 46 %) and sorafenib + everolimus combination (4-month PFS > 55 %) [8, 9].

The response rate for GD was 13 % (17 % if we exclude HGS). With HDIFO, the treatment of choice in 1st line for metastatic osteosarcoma, response rates described range between 10 and 62 % [6, 20, 21], while for other drugs such as cyclophosphamide and etoposide, objective responses were seen in 19 % and 28.5 % of patients respectively in different studies [22, 23]. With sorafenib, as monotherapy, or in combination with m-TOR inhibitors, the response rate is very low (sorafenib: 8 %, sorafenib + everolimus: 5 %) [8, 9].

There are few published data assessing the activity of GD in patients with recurrent osteosarcoma. Conflicting results are reported and most of the studies were based on a small number of patients (Table 1; [13–18]).

In a study by Song et al., a response rate of 24 % was described in 17 pediatric patients (3 CR and 1 PR) [18]. The median OS was 9 months (range, 0.6–79.6) [18], similarly to the median OS of the present study (7.5 months, range 2–45).

On the contrary, the prospective SARC (Sarcoma Alliance for Research Through Collaboration) study exploring the activity of GD in a group of different bone sarcomas (including Ewing, chondrosarcoma and 14 patients with osteosarcoma), demonstrated a lack of activity in all cohorts, including osteosarcoma [15].

In our series, grade 3–4 neutropenia or thrombocytopenia was observed in 25 % of the patients, with no difference according to gemcitabine dose and with only 1 patient with febrile neutropenia, which was described in about 14 % of cycles after HDIFO [24]. More importantly our study confirms that taxane-related hypersensitivity reactions (HSR) can be an issue: 3 (6 %) patients experienced allergic reactions during docetaxel infusion (two following the first cycle and one after 4 cycles), and were subsequently treated with gemcitabine monotherapy; in the other 2 pediatric cases, grade 3 Stephen Johnson syndrome and capillary leak syndrome were documented, in one patient each (Table 4). Taxane-related HSR usually occurs within the first minutes of infusion in up to 30 % of patients, without premedication, and ≤4 % with antihistamine and steroid premedication. This is dose and rate-dependent, and adequate premedication and patient monitoring on commencing treatment is recommended [25].

Compared to other therapeutic options in this setting, we did not observe renal toxicity. This can be an issue with HDIFO in about 25 % of cases, and in half of these, irreversible renal failure can occur [24].

Unlike sorafenib, which can cause pneumothorax and permanent treatment discontinuation in about 3 % of patients [8, 9], gemcitibine and docetaxel-induced tumor-shrinkage, was not associated with pneumothorax in our series.”

## Conclusion

In conclusion, there are few new active regimens for patients with relapsed osteosarcoma following multimodality therapy [26]. To our knowledge, this is the largest series of patients treated with GD for relapsed bone sarcoma published. While a prospective SARC phase II study showed marginal activity for GD in metastatic osteosarcoma [15], partly due to limitations in clinical trial design, as suggested by the authors themselves [15], we believe that the combination is active and should be included in the therapeutic armamentarium of metastatic osteosarcoma.

## Abbreviations

CTCAE: Common Toxicity Criteria for Adverse Events
D: docetaxel
G: gemcitabine
HGS: high-grade spindle cell sarcoma of bone
OS: overall survival
PD: progressive disease
PFS: progression-free survival
PR: partial response
RECIST: Response Evaluation Criteria In Solid Tumors
sCR2: surgical complete response
SD: stable disease
